# Pain and sickness behavior associated with corneal lesions in dairy calves

**DOI:** 10.12688/f1000research.6649.1

**Published:** 2015-08-11

**Authors:** Brandon J. Woods, Suzanne T. Millman, Natalia A. da Silva, Reneé D. Dewell, Rebecca L. Parsons, Chong Wang, Annette M. O'Connor

**Affiliations:** 1 Veterinary and Diagnostic Production Animal Medicine, College of Veterinary Medicine, Iowa State University, Ames, IA, 50011, USA; 2 Department of Biomedical Sciences, College of Veterinary Medicine, Iowa State University, Ames, IA, 50011, USA; 3 Center for Food Security and Public Health, College of Veterinary Medicine, Iowa State University, Ames, IA, 50011, USA; 4 Department of Statistics, College of Liberal Arts and Sciences, Iowa State University, Ames, IA, 50011, USA

**Keywords:** calves, infectious bovine keratoconjunctivitis, ocular pain, sickness behavior

## Abstract

Infectious bovine keratoconjunctivitis (IBK) is a common corneal disease of calves that adversely affects animal welfare by causing pain and weight loss. Identifying behavioral indicators of pain and sickness in calves with IBK is necessary for designing studies that aim to identify effective means of pain mitigation. Consistent with principles of the 3Rs for animal use in research, data from a randomized blinded challenge study was used to identify and describe variation of behaviors that could serve as reliable indicators of pain and sickness in calves with corneal injuries. Behavioral observations were collected from 29 Holstein calves 8 to 12 weeks of age randomly allocated to one of three treatments: (1) corneal scarification only, (2) corneal scarification with inoculation with *Moraxella bovoculi *and (3) corneal scarification with inoculation with *Moraxella bovis*. Behavior was continuously observed between time 1230 - 1730 h on day -1 (baseline time period) and day 0 (scarification time period). Corneal scarification and inoculation occurred between 0800 - 1000 h on day 0. Frequency of head-directed behaviors (head shaking, head rubbing, head scratching) and durations of head rubbing, feeding, standing with head lifted, lying with head lifted and sleeping were compared between study days and groups. Following scarification, the frequency of head-directed behavior significantly increased (p = 0.0001), as did duration of head rubbing (p=0.02). There was no significant effect of trial, trial day, treatment or treatment-day interaction on other behaviors studied. Our study demonstrated that head-directed behavior, such as head shaking, rubbing and scratching, was associated with scarification of eyes using an IBK challenge model, but sickness behavior was not observed.

## Preamble

The authors affirm that this manuscript is an honest, accurate, and transparent account of the study being reported; that no important aspects of the study have been omitted; and that any discrepancies from the review as planned have been documented and explained. The authors have indicated where results from these study animals are reported in other publications, and have including citations for these publications where relevant.

## Introduction

Infectious bovine keratoconjunctivitis (IBK) is a disease of cattle, causing corneal edema and ulceration, photophobia, blepharospasm and ephiphora ( Gelatt, 2008; George, 1984). IBK can occur in 20–30% of calves in a beef calf crop, with an estimated 30% of beef herds affected annually ( Brown *et al.*, 1998). Incidence of IBK has been associated with 6.8 to 13.6 kg decreased weaning weight ( Funk *et al.*, 2009; Snowder *et al.*, 2005). As there is evidence that vaccination is an ineffective strategy in isolation ( Funk *et al.*, 2009; Kizilkaya *et al.*, 2013; O'Connor *et al.*, 2012); producers must identify non-responding animals and minimize the impact of IBK once diagnosed with antibiotic treatment ( O'Connor *et al.*, 2006).

Blepharospasm and photophobia suggest IBK is painful ( Williams, 2010) and pain mitigation therapies may be useful adjuncts to antibiotic therapy by improving animal welfare and reducing weight loss. Since blepharospasm, photophobia and ocular discharge are the earliest indications of IBK ( Postma *et al.*, 2008), suggesting that detection occurs only once the condition is quite advanced. In livestock and horses, ocular injury can occur as a result of irritation of the corneal surface by dust, tall grasses, weeds or contact with other elements in the environment, such as fencing. Mechanical injury to the eye increases susceptibility of cattle to IBK infection ( Postma *et al.*, 2008), and identification of behavior responses to injury may provide opportunity for early detection of corneal injury and preventive treatment. Although subjective scoring of behavior associated with acute IBK infection has been described in the literature as an aspect of signalment and clinical assessment, scientific investigation of behavioral responses to ocular injury and infection is needed.

We have postulated that pain and sickness behavior associated with IBK might reduce nursing and forage consumption and explain the weight loss commonly associated with this disease. Cattle display behavioral changes in response to pain that may be specific to the nature of the injury ( Millman, 2013). For example, calves display significant increases in ear flicking and head rubbing behaviors ( Duffield *et al.*, 2010; Faulkner & Weary, 2000; Heinrich *et al.*, 2010), that occur concurrently with increases in physiological and biochemical responses ( Heinrich *et al.*, 2009) following cautery disbudding surgery, and which are mitigated by postsurgical analgesia with nonsteroidal anti-inflammatory drugs. Further, the cytokine cascade associated with the inflammatory response evokes characteristic “sickness behaviors”, such as anorexia and increased rest ( Millman, 2007; Watkins & Maier, 2000). Associations between morbidity and changes in feeding and social behavior have been identified in cattle ( Weary *et al.*, 2009). However, a paucity of information about pain and sickness behavior specifically associated with IBK or ocular insults presents barriers to testing our hypothesis.

Previous research by our team suggests that mechanical ocular injury is painful to calves ( Dewell *et al.*, 2014). A randomized and blinded disease challenge study was conducted to assess putative causal organisms for IBK incidence in calves ( Gould *et al.*, 2013). A mechanical ocular injury was administered in one eye (“scarification”), followed by inoculation with *Moraxella bovis (M. bovis), Moraxella bovoculi (M. bovoculi)* or no inoculation. Only calves in the *M. bovis* treatment developed IBK-associated corneal abnormalities. Concurrent with the microbiological study, we evaluated clinical approaches for qualifying ocular pain in calves, using pressure algometry, a Cochet Bonet aesthesiometer, blepharospasm and photophobia ( Dewell *et al.*, 2014). Significant changes in mechanical nociception threshold scores were observed following scarification relative to baseline values prior to treatment suggesting increased pain sensitivity, but neither IBK inoculation nor corneal ulceration were associated with differences in nociception responses. Retrospective video analysis of the calves enrolled in this study presented a unique opportunity for detailed investigation of behavioral changes in the home pen associated with ocular injury and IBK infection.

The objective of this study was to maximize the value of data obtained from a prior study by describing the magnitude and variation of pain and sickness behaviors in calves with experimental induced ocular injury and infection. Such information will facilitate early detection of affected animals by animal caregivers and veterinarians. Furthermore, this information will enable researchers to appropriately design studies to assess the effectiveness of pain mitigation strategies and design studies to assess the extent to which pain and sickness behaviors contribute to weight loss associated with IBK.

## Materials and methods

### Study location and study population

This study is a hypothesis generating study and represents a secondary use of animals enrolled in an experimental study conducted to assess putative causal organisms for IBK in calves ( Gould *et al.*, 2013). This experimental study population provided a unique controlled setting for pain and sickness behaviors in calves with corneal scarification and IBK. Three trials (replicates) were conducted in January 2012 (Trial 1), May 2012 (Trial 2) and August 2012 (Trial 3). Dairy breed calves, predominantly Holstein genetics with some Jersey influence, and 8 to 12 weeks of age, were sourced from the Iowa State University (ISU) Dairy Farm (Trial 1 and Trial 2) and a private Iowa-based owner (Trial 3). Calves were housed in a biosecurity Level 3 facility at ISU in Ames, Iowa. For each trial, all enrolled calves were housed in a single room maintained at 20–21°C (68–70°F). Each calf was housed separately in raised 0.9 × 1.8 meter (3 × 6 foot) pens that provided no opportunity for calf-to-calf contact. Auditory contact among calves was not restricted, and visual contact among calves was limited to the unique position and location of each pen. Calves were provided free choice water and were fed mixed grass hay and a pre-mixed calf starter (Heartland Co-op, Des Moines, IA). To avoid cross contamination, caretakers and research personnel wore protective gloves and clothing when working with the calves. If personnel had physical contact with calves during animal husbandry and study related activities, protective items were changed before a new calf or equipment or facilities associated with another calf was contacted. Other biosecurity measures included providing separate feeders and individual automatic watering troughs. Approval for this study was obtained from the Iowa State University (ISU) Institutional Biosafety Committee (IBC#11-D-0017-A) and the Institutional Animal Care and Use Committee (IACUC 8-11-7187-B).

### Enrollment of animals

Prior to enrollment in each replicate on day - 4, calves received an extensive ophthalmic examination by a board certified veterinary ophthalmologist and a supervised veterinary ophthalmology intern as described previously ( Gould *et al.*, 2013). Only calves without identified ocular abnormalities on day - 4 were enrolled.

### Sample size

The sample size for the original study was based on estimated IBK risk of infection between groups. The study enrolled 36 calves to obtain 80% power to detect an estimated 60% difference in risk between groups based on an expected 10% IBK risk in controls and at least 70% IBK risk in inoculated animals with significance level 0.05. It was not possible to calculate the power of the study to detect meaningful differences in pain or sickness behavior outcomes as estimates of normal levels or normal variation associated with IBK were not available prior to the study.

### Treatment description and allocation

Upon enrollment on day - 4, calves were allocated to one of three treatments using a random number generator (Microsoft Excel, 2007) and then the left or right eyes were randomly allocated for corneal scarification. The three treatments were:

a. 1) corneal scarification only (control)
b. 2) corneal scarification with inoculation with *Moraxella bovoculi (*ATCC strain: BAA- 1259; Origin: California, depositor: Dr. J Angelos) ( *M. bovoculi*)
c. 3) corneal scarification with inoculation with *Moraxella bovis* (strain Epp63-300; Dr. Rosenbusch lab; Origin: National Animal Disease Center) ( *M. bovis*)

A concurrent negative control treatment was not relevant to the question posed by the original experiment therefore for the question assessed by this study in lieu of a concurrent negative control treatment it was necessary we used the response prior to scarification to represent behaviors expected under the no pain condition.

Corneal scarification took place between 0800–1000 h on day 0. Only one eye from each calf was scarified. Scarification was accomplished by a researcher trained in the procedure and according to a published protocol ( Rosenbusch & Ostle, 1986). Calves were individually restrained using a portable modified head restraint placed on the front of each pen. Prior to scarification, the cornea of one eye of each calf was anesthetized 3–5 minutes prior to the scarification procedure with topical 0.5% proparacaine hydrochloride (Bausch & Lomb Inc., Rochester NY). A sterilized wire brush approximately 5 mm in length was used to create 3–4 horizontal and vertical superficial epithelial scratches. To inoculate eyes with *M. bovoculi* or *M. bovis*, a sterile swab was rolled across a blood agar plate containing the organism. For each scarified eye, the swab was rolled or wiped across the cornea as well as introduced into the medial conjunctiva sac. To blind the allocation status of each eye, the researcher preparing the swabs concealed the allocation status from the researcher conducting the scarification procedure. Calves were restrained and observed for development of centrally located corneal ulcerations consistent with IBK on days +1, 3, 6, 8 and 10 relative to scarification. The results of the original microbiological causation study ( Gould *et al.*, 2013) and the mechanical nociception thresholds ( Dewell *et al.*, 2014) are published elsewhere. If a corneal ulcer was identified and reached 15mm diameter or wider, the calf was euthanized on the same day using an appropriate dose of sodium pentobarbital administered intravenously by or under the supervision of a licensed veterinarian. At the conclusion of the study, all remaining calves were euthanized using an appropriate dose of sodium pentobarbital administered intravenously by or under the supervision of a licensed veterinarian.

### Behavioral recording

Calf behavior was recorded using digital video recording. Video images were captured, utilizing three Noldus portable labs (Noldus Information Technology, Wageningen, NL), one for each cohort of four calves. One color Panasonic camera (WV-CP484, Kadoma, Japan) was mounted above each stall, and positioned to ensure maximum stall and calf visibility. The 12 cameras were divided into three zones, one for each Noldus video portable lab, based on the location of the stalls. Every zone contained four cameras each directed at a specific stall and fed into a multiplexer, which allowed the image to be recorded onto a PC using HandiAvi (v4.3, Anderson’s AZcendant Software, Tempe, AZ) at 30 frames per second. Color video with no audio was continuously recorded between 0500–2000 h from day -1 to day 10 relative to scarification. However, due to the rapid development of IBK ulcers in one scarification treatment group, behavior outcomes were collected from video recordings on day -1 and on day 0 after the scarification procedure only.

### Data collection

Behavioral observations were collected by a single trained technician (BW) from 22 May 2013 until 3 July 2013, using Observer® (v10.1.548, Noldus Information Technology, Wageningen, NL). This person was unaware of the actual treatments the calves received and the allocation groups until after all data were collected and the preliminary data analysis conducted. The individual was aware that the study related to ocular lesions. Prior to data collection, an ethogram was developed by two co-authors (SM and RP). The behaviors were selected based on prior behavior studies involving pain and sickness behaviors ( Duffield *et al.*, 2010; Millman, 2007; Todd *et al.*, 2007; Millman, 2013). Table 1 describes the ethogram. Blink rates and eye movements (open, closed, fixed and rolled) would be relevant behaviors associated with ocular pain; however, these behaviors were not included because the eyes of the calves could not be reliably seen throughout the duration of the video. Ear-flicking was omitted based on similar logic. Frequencies were recorded for behavioral events whereas durations were recorded for behavioral states. An event is a behavioral pattern of relatively short duration, and in our study independent events were separated by five or more seconds. In contrast, a state is a behavioral pattern of a longer duration such as posture or prolonged activity ( Martin & Bateson, 2007).

**Table 1.** Ethogram for observing recorded video using Observer® (v10.1.548, Noldus Information Technology, Wageningen, NL) software for all trials and treatments during baseline and scarification time periods.

Behavior Name	Modifier	Description	Outcome variable
Head scratching		Calf scratches head with its right or left pelvic limb digit	Frequency
Head shaking		Calf moves head rapidly back and forth for more than two repetitions	Frequency
Feeding		Head is positioned over or inside of the feed bunk for two or more seconds	Duration
Standing	Head lifted	Calf is standing on all four feet with head lifted, supported by the neck and in motion; includes movement around pen	Duration
	Licking	Calf is standing with head turned to either right or left side and licking body	Duration
	Head rubbing	Calf is standing and rubbing head on body or part of the pen	Duration and Frequency
	Out of view	Calf is standing, but head cannot be seen	Duration
Lying	Head lifted	Calf is positioned in ventral recumbency with legs tucked under the body and head raised or in motion, head not shaking	Duration
	Head tucked right	Calf is positioned in ventral recumbency with legs tucked under the body and head tucked to the right side of body, head is motionless	Duration
	Head tucked left	Calf is positioned in ventral recumbency with legs tucked under the body and head tucked to the left side of body, head is motionless	Duration
	Head in sternal recumbency	Calf is positioned in ventral recumbency with legs tucked under the body and head positioned ventrally on ground, the head is not tucked towards the body, head is motionless	Duration
	Head rubbing	Calf is positioned in ventral recumbency with legs tucked under the body while rubbing head on body or part of the pen	Duration and Frequency
	Licking	Calf is positioned in ventral recumbency with legs tucked under the body while licking part of its body	Duration
	Out of view	Calf is positioned in ventral recumbency with legs tucked under the body, but its head cannot be seen	Duration
Drinking		The calf’s head is positioned over the watering trough for more than two seconds or the calf is visibly drinking from the watering trough	Frequency
Disturbance		Calf is disturbed by a human entering its pen or standing in front of pen, by human cleaning in or around pen, or by human collecting data	Duration
Other		Any other behavior not captured in ethogram	Duration

Research technician training on the Observer® program and the ethogram comprised two weeks, and data collected was compared relative to data collected by a trained research associate with several years of experience (RP). Behavioral data collection formally began when the inter-observer reliability values were: proportion of agreements = 0.72, Kappa = 0.68, Rho = 0.98. Intra-observer reliability was checked periodically throughout the video observation period and were: proportion of agreements = 0.79, Kappa = 0.78, and Rho = 0.98.

**Figure 1.**
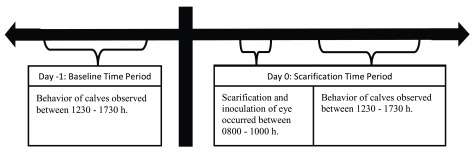
Timeline of study.

Behavioral data was collected using continuous sampling of hour segments of video recorded from 1230–1730 on day -1 (hereafter referred to as baseline time period) and on day 0 (hereafter referred to as scarification time period). See Figure 1 for timeline of study. These days of interest were selected to enable identification of changes in behavior associated with the scarification treatments when the full cohort of calves was present in each treatment. The time of day, 1230–1730 h, was selected because calves were least likely to be disturbed by caretakers or research personnel during this time period, there was sufficient light to enable detailed behavior observations and to accommodate the timing of the scarification procedure (0800–1000 h).

Prior to data collection, video was cut using the Virtual Dub® software (Avery Lee, compiled with Microsoft Visual Studio 2005 for X86, version 1.9.9) into approximately 90 minute blocks to facilitate blinding of the observer to treatment, day and time. The order of video blocks observed was randomized using a random number generator in MS Excel®. Calves were observed in groups of four in real time and frequencies and durations according to the ethogram were recorded. The observer took periodic 5–10 minute breaks after observing 90 minutes of video.

### Descriptive analysis

The frequencies of head shaking, head scratching, and drinking over the five-hour period for baseline and scarification time periods were calculated as categorical variables. The durations for all other behaviors were calculated as continuous variables in minutes for the total observed time for each day over the five-hour period. For each behavior, the distribution was evaluated using visual examination of a box plot and presented based on current recommendations for reporting ( Lang & Altman, 2013). After this preliminary analysis and data check, the researchers were unblinded to the treatments.

### A Priori hypotheses

The results of an analysis of the pressure algometry data suggested that the calves experienced increased pain sensitivity after scarification as indicated by reduced mechanical nociception thresholds ( Dewell *et al.*, 2014). However, since only calves in the *M. bovis* treatment developed IBK-ACA ( Gould *et al.*, 2013), we hypothesized these calves would show sickness behavior associated with inflammation and proinflammatory cytokines. These observations lead to the *a priori* hypotheses (i.e. before the data were unblinded) that pain related behaviors should differ between baseline time period and scarification time periods for all calves. We also hypothesized that sickness behavior would occur in calves enrolled that received *M. bovis,* since these calves subsequently developed IBK, but would not occur in calves enrolled in the *M. bovoculi* or control treatments since these calves did not develop IBK. The statistical analysis were designed *a priori* to test these hypotheses.

### Data transformations and manipulations

Behaviors observed that were expected to be important indicators of ocular pain (or irritation) were head-directed behaviors: head shaking, head rubbing and head scratching. The frequencies of these three head-directed behaviors (head scratching, head rubbing, and head shaking) were summed to create a variable that described the total frequency of any head-directed behavior. Similarly, although measured separately in the ethogram, the behavioral states standing and head rubbing and lying and head rubbing were combined into a single state termed head rubbing for analyses purposes. Head rubbing was a unique behavior of interest because we considered it both an event and a state so its frequency and duration were calculated.

Behaviors in the ethogram that were expected to be important indicators of sickness behaviors were feeding, standing with head lifted, lying with the head lifted, and sleeping. Lying with head in sternal recumbency and lying and licking were recorded, but were rare and removed from further analysis. The behaviors lying with the head tucked to the left and lying with the head tucked to the right were observed separately, but were later combined for the analyses and are described in this study as sleeping based on previous research and simplicity ( Ternman *et al.*, 2012).

### Hypothesis testing for analysis for pain behaviors

For all analyses the unit of analysis was the calf. All models were executed using PROC GLIMMIX (SAS 9.3, Inst. Inc., Cary, NC). Explanatory variables for all models were treatment (three scarification treatments), time period (two time periods prior to and following scarification i.e. baseline and scarification) and trial, which refers to the three trials conducted. All models included the fixed effects treatment, time period and the interaction between treatment and time period. Random effects were trial and animal. When main effects were significant based on a p value < 0.05, least square means were calculated and the differences between means were tested using Turkey-Kramer adjustment for multiple comparison when appropriate.

For head-directed behavior, two potential generalized linear models were assessed i.e., Poisson and negative binomial. A scaled Pearson’s statistic ( $\frac{\chi_{n}^{2}}{df}$ where *df* is the degrees of freedom) was used to select the preferred modeling approach. We preferred the model with the statistics closer to 1. The negative binomial was chosen.

Count data were analyzed with mixed effect negative binomial regression models, with treatment, time and interaction between treatment and time as fixed effects and animal nested in treatment and trial as random effects. Logarithm of total time of observation was used as offset in the negative binomial models.

For behaviors in which duration was calculated (head rubbing, feeding, standing with head lifted, lying with head lifted and sleeping), duration data were log transformed and analyzed with linear mixed models, with treatment, time and interaction between treatment and time as fixed effects and animal nested in treatment and trial as random effects. Logarithm of total time of observation was used as offset.

Fixed effects were not excluded based on non-significance. Diagnostic tests were conducted to check the model assumptions for all models. In addition, the normality assumptions and alternative models were checked. The akaike information criterion (AIC) criteria or likelihood ratio tests (LRT) were used to select the final model when the models were nested.

## Results

### Study population

Of the 36 animals purchased for the study, five calves were ineligible for enrollment due to pre-existing corneal abnormalities. Thirty-one enrolled calves were randomly allocated to the three treatments over the three trials, resulting in uneven numbers of calves per treatment: scarification only (n = 11), scarification and inoculation with *M. bovoculi* (n = 10), scarification and inoculation with *M. bovis* (n = 10). Behavior data from two calves enrolled in trial 3 were missing due to camera malfunctions. These missing data were from one calf in the *M. bovis* treatment and one calf in the *M. bovoculi* treatment. Therefore data for this study included scarification only (n = 11), scarification and inoculation with *M. bovoculi* (n = 9) and scarification and inoculation with *M. bovis* (n = 9). Details from results of other analysis associated with the study are available elsewhere ( Gould *et al.*, 2013).

Based on visual assessment of the data, neither event nor state behaviors were normally distributed, therefore the data were reported as median, minimum, 25 ^th^ quartile, 75 ^th^ quartile and maximum values, compliant with current statistical reporting guidelines ( Lang & Altman, 2013). Table 2 reports this summary information for event behaviors. Because drinking events were observed, but were too rare to be relevant to the hypothesis they are reported here in text. For drinking events, the median (minimum, 25 ^th^ percentile, 75 ^th^ percentile, maximum) during baseline time period for control, *M. bovoculi* and *M. bovis* treatments were 4.0 (0.0, 1.5, 6.5, 17.0), 3.0 (0.0, 2.0, 5.0, 9.0), and 4.0 (1.0, 2.0, 5.0, 6.0) respectively. The median (minimum, 25 ^th^ percentile, 75 ^th^ percentile, maximum) during scarification time period for control, *M. bovoculi* and *M. bovis* treatments were 4.0 (1.0, 3.0, 7.0, 11.0), 6.0 (2.0, 4.0, 7.0, 13.0), and 4.0 (2.0, 3.0, 6.0, 9.0) respectively.

**Table 2.** Median (minimum, 25 ^th^ percentile, 75 ^th^ percentile, maximum) values of frequency of events for head shaking, head rubbing and head scratching by treatment over five hour observation periods during baseline and scarification time periods (n = number of calves observed).

Day	Behavior	Scarification only (n = 11)	Scarification and inoculation with *M. bovoculi* (n = 9)	Scarification and inoculation with *M. bovis* (n = 9)
**Day -1 (baseline time period)**				
	Head shaking	0.0 (0.0, 0.0, 2.0, 4.0)	0.0 (0.0, 0.0, 0.0, 2.0	1.0 (0.0, 1.0, 2.0.,4.0)
	Head rubbing	4.0 (0.0, 2.5, 7.5, 14.0)	1.0 (0.0, 0.0, 4.0, 14.0)	4.0 (2.0, 3.0, 5.0, 9.0)
	Head scratching	0.0 (0.0, 0.0, 1.5, 6.0)	0.0 (0.0, 0.0, 0.0, 4.0)	0.0 (0.0, 0.0, 1.0, 2.0)
**Day 0 (scarification time period)**				
	Head shaking	1.0 (0.0, 1.0, 5.0, 47.0)	2.0 (0.0, 1.0, 3.0, 47.0)	5.0 (0.0, 2.0, 13.0, 43.0)
	Head rubbing	4.0 (0.0, 1.5, 7.5, 18.0)	7.0 (4.0, 6.0, 11.0, 37.0)	14.0 (1.0, 2.0, 18.0, 27.0)
	Head scratching	1.0 (0.0, 0.5, 3.0, 18.0)	2.0 (0.0, 1.0, 3.0, 4.0)	2.0 (0.0, 1.0, 4.0, 15.0)

The descriptive statistics for behavioral states during baseline and scarification time periods are reported in Table 3 and Table 4 respectively. All behaviors listed in the ethogram were observed in calves in each treatment during both time periods. Lying with the head lifted and feeding were the two most commonly recorded behaviors. Lying in sternal recumbency, lying and head rubbing, and lying and licking were very rare behaviors. The maximum disturbance time for all trials was 6.5 minutes; however the median disturbance time for all trials during the baseline time period and scarification time period was 0. Standing out of view was an extremely rare observation (median for all treatments = 0), and lying out of view was never observed.

**Table 3.** Median, (minimum, 25 ^th^ quartile, 75 ^th^ quartile, maximum) values for duration of behavioral states in minutes by treatment during baseline time period (n = number of animals).

Day -1 (Baseline time period)	Scarification only (n = 11)	Scarification and inoculation with *M. bovoculi* (n = 9)	Scarification and inoculation with *M. bovis* (n = 9)
Standing with head lifted	14.2 (2.5, 8.15, 26.7, 46.0)	15.8 (3.0, 5.4, 29.7, 73.5)	21.1 (12.2, 16.5, 24.2, 41.3)
Standing and head rubbing	1.8 (0.0, 0.90, 2.8, 6.8)	0.5 (0.0, 0.0, 2.3, 6.2)	1.9 (0.5, 1.0, 2.8, 5.5)
Standing and licking	3.9 (0.8, 2.1, 4.6, 13.8)	2.1 (0.4, 1.3, 3.4, 7.0)	3.4 (1.0, 2.6, 4.4, 7.3)
Lying with head lifted	149.7 (106.3, 134.1, 159.7, 197.8)	157.3 (88.1, 140.5, 165.8, 191.7)	176.7 (124.1, 170.3, 189.4 237.6)
Sleeping	22.8 (0.3, 14.4, 43.9, 76.0)	34.1 (8.9, 27.9, 44.2, 77.8)	29.3 (8.9, 14.7, 49.7, 62.7)
Lying with head in sternal recumbency	1.3 (0.0, 0.0, 6.4, 12.0)	0.0 (0.0, 0.0, 0.0, 0.9)	0.0 (0.0, 0.0, 2.1, 12.6)
Lying and head rubbing	0.0 (0.0, 0.0, 0.1, 0.3)	0.0 (0.0, 0.0, 0.0, 0.0)	0.0 (0.0, 0.0, 0.0, 0.0)
Lying and licking	0.9 (0.0, 0.6, 3.4, 8.4)	4.3 (0.0, 0.4, 5.2, 10.7)	2.4 (0.6, 1.7, 4.6, 5.7)
Feeding	47.9 ( 16.7, 36.9, 87.8, 107.1)	39.2 (6.0, 30.4, 73.1, 108.8)	52.1 (13.3, 49.2, 72.1, 84.8)
Out of view standing	0.0 (0.0, 0.0, 0.0, 0.0)	0.0 (0.0, 0.0, 0.0, 0.0)	0.0 (0.0, 0.0, 0.0, 0.6)
Disturbance	0.0 (0.0, 0.0, 0.7, 6.3)	0.0 (0.0, 0.0, 0.7, 6.5)	0.0 (0.0, 0.0, 0.8, 6.5)
Total time observed	298.2 (167.4, 264.2, 299.1, 299.5)	298.3 (167.4, 291.0, 299.1, 299.6)	298.4 (291.0, 296.1, 298.5, 299.6)

**Table 4.** Median, (minimum, 25 ^th^ quartile, 75 ^th^ quartile, maximum) values for duration of behavioral states in minutes by treatment during scarification time period (n = number of animals).

Day 0 (Scarification time period)	Scarification only (n=11)	Scarification and inoculation with *M. bovoculi* (n=9)	Scarification and inoculation with *M. bovis* (n=9)
Standing with head lifted	26.3 (7.8, 14.3, 45.9, 54.8)	29.2 (12.2, 26.1, 34.3, 40.9)	29.5 (18.8, 22.2, 33.8, 48.6)
Standing and head rubbing	0.9 (0, 0.5, 2.2, 9.7)	2.7 (2.0, 2.4, 3.1, 13.2)	3.8 (0.4, 0.9, 10, 13.8)
Standing and licking	3.5 (1.9, 3.1, 5.2, 13.7)	5.2 (1.8, 2.9, 6.4, 11.2)	4.5 (0.2, 4, 5.8, 15.8)
Lying with head lifted	171.9 (124.5, 143.5, 177.1, 187.5)	160.5 (124.2, 147.4, 199.6, 214.8)	161.5 (135.2, 136.5, 184.4, 247.6)
Sleeping	27 (0, 21.5, 44.1, 66.4)	16.3 (0, 10.9, 34.5, 62.8)	28.9 (0, 8.6, 44.9, 83.7)
Lying with head in sternal recumbency	0 (0, 0, 1.4, 30.1)	0 (0, 0, 0, 4.5)	0 (0, 0, 0, 3.3)
Lying and head rubbing	0 (0, 0, 0, 0.7)	0 (0, 0, 0.3, 10.4)	0 (0, 0, 0.2, 1.5)
Lying and licking	2.5 (0.1, 1.4, 5.6, 6.8)	1.9 (0, 0.4, 5.7, 10.1)	1.2 (0, 0.7, 4, 19)
Feeding	48.5 (23.2, 37.1, 74, 106.4)	60.8 (29.2, 44.4, 89.9, 90.6)	51 (6.2, 27.9, 65.2, 97.8)
Out of view standing	0 (0, 0, 0, 0.5)	0 (0, 0, 0, 0 )	0 (0, 0, 0, 0)
Disturbance	0 (0, 0, 2.3, 5.8)	0.2 (0, 0, 3.2, 3.6)	0.3 (0, 0, 3.2, 3.9)
Total time observed	298.1 (293.2, 296.5, 298.7, 300.0)	296.7 (294.6, 295.7, 298.1, 300.0)	296.1 (294.8, 296.1, 297.6, 298.2)

### Hypothesis testing differences in head-directed pain behaviors

The frequencies of the three separate head-directed behaviors were too rare to enable modeling. Consequently, we combined the three separate head-directed behaviors into one measure, and the results of the analysis of the combined head-directed outcome are reported. No significant interaction between treatment and time period was observed (p = 0.18). However, the effect of time period was significant (p = 0.0001), whereas the effect of treatment was not significant (p = 0.42). The least squares means comparison of the baseline time period and scarification time period was not significant for the control treatment (p = 0.75). However, it was significantly different for the *M. bovocul*i group (p value = 0.02) and the *M. bovis* group (p = 0.04). Table 5 shows the regression-based estimates and 95% confidence interval of the head-directed behavior frequencies for the three treatments during the baseline time period and scarification time period. These estimates can be used as a basis for future sample size determination. The random effect for animal was (0) and the estimate and standard error for trial term were 0.21 and 0.25 respectively.

**Table 5.** Estimates (95% confidence interval) in log units of all six models of pain and sickness behaviors for the three treatments during baseline and scarification time periods. Head-directed behaviors are events in units of log of link. Head-directed behaviors back transformed are in units of frequency of event per hour. Head rubbing, feeding, standing with head lifted, lying with head lifted and sleeping are behavioral states in units of log of proportion.

Variable	Time Period	Scarification only (n=11)	Scarification and inoculation with *M. bovoculi* (n=9)	Scarification and inoculation with *M. bovis* (n=9)
Head-directed behaviors ^a^	Baseline time period	-3.6 (-4.4, 2.8)	-4.3(-5.1, -3.4)	-3.7 (-4.6, -2.9)
	Scarification time period	-3.1 (-3.9, -2.3)	-2.7 (-3.5, -1.9)	-2.4 (-3.2, -1.6)
Head-directed behaviors	Baseline time period	1.6 (0.7, 3.6) ()	0.8 (0.3, 2.0) ()	1.4 (0.6, 3.3) 0.6
back transformed ^b^	Scarification time period	2.8 (1.3, 6.0)	4.1(1.8, 9.2)	5.5 (2.4, 12.3)
Head rubbing ^c^	Baseline time period	-5.7 (-6.7, -4.6)	-6.8 (-8.0, -5.6)	-5.1 (-6.4, -4.0)
	Scarification time period	-5.6 (-6.7, -4.6)	-4.4 (-5.6, -3.3)	-4.5 (-5.6, -3.3)
Feeding ^c^	Baseline time period	-1.7 (-2.1, -1.2)	-2.1 (-2.5, -1.6)	-1.9 (-2.3, -1.4)
	Scarification time period	-1.8 (-2.2, -1.3)	-1.6 (-2.1, -1.2)	-2.1 (-2.5, -1.6)
Standing with head lifted ^c^	Baseline time period	-3.0 (-3.5, -2.5)	-3.0 (-3.5, -2.4)	-2.7 (-3.2, -2.1)
	Scarification time period	-2.5 (-3.0, -2.0)	-2.4 (-3.0, -1.9)	-2.4 (-2.9, -1.8)
Lying with head lifted ^c^	Baseline time period	-0.6 (-0.7, -0.5)	-0.6 (-0.7, -0.5)	-0.5 (-0.6, -0.4)
	Scarification time period	-0.6 (-0.7, -0.5)	-0.6 (-0.7, -0.5)	-0.6 (-0.7, -0.5)
Sleeping ^c^	Baseline time period	-2.6 (-3.8, -1.4)	-2.0 (-3.3, -0.7)	-2.4 (-3.7, -1.1)
	Scarification time period	-3.0 (-4.1, -1.7)	-3.4 (-4.7, -2.1)	-3.2 (-4.5, -1.8)

^a^ = units (log of link)

^b^ = units (frequency of event per hour)

^c^ = units (log of proportion)

The original proposed model for duration of time spent head rubbing, with trial included as a random effect, did not converge. Therefore, the model was modified to include trial as a fixed effect rather than a random effect. The p values for trial, time period, treatment and time period by treatment interaction were 0.65, 0.02, 0.23, and 0.08 respectively. The duration of time spent head rubbing did not differ significantly between baseline time period and scarification time period for the control treatment (p=1) and for the *M. bovis* treatment (p=0.92). However, duration of time spent head rubbing was significantly increased after scarification for the *M. bovoculi* treatment (p = 0.041). The variance component for the random effect and its standard error for animal were 0.36 and 0.58 respectively.

### Hypothesis testing differences in sickness behaviors

In no model was there a significant difference among treatments or between time periods for the total duration spent feeding, standing with head lifted, lying with head lifted or sleeping. The model-derived estimates of the transformed data are reported in Table 5. These estimates may inform future study design.

For the feeding outcome, the p values for the fixed effects were the following for time period, treatment and time periods by treatment interaction: 0.62, 0.56 and 0.09. The random effect variance estimate and its standard error for animals were 0.21 and 0.09 respectively. The random effects estimate and standard error for trial were 0.01 and 0.04 respectively.

For the standing with head lifted outcome, the p values of the fixed effects for time period, treatment and time period by treatment interaction were 0.01, 0.55 and 0.81 respectively. The random effects estimate and standard error for animal were 0.06 and 0.09 respectively. The random effects for estimate and standard error for trial were 0.07 and 0.09 respectively.

For the lying with head lifted outcome, trial was calculated as a fixed effect. The model estimate of the p value for the fixed effects for trial, time period, treatment and time periods by treatment interaction were 0.92, 0.37 and 0.76 and 0.69 respectively. The random effect variance estimate and its standard error for animal were 0.02 and 0.01 respectively.

For the sleeping outcome, the p values for the fixed effects time period, treatment and time period by treatment interaction were 0.09, 0.99, and 0.63 respectively. The estimate and standard error of the variance component for animal were 0.19 and 0.69 respectively. The random effect variance estimate and its standard error for time period were 0.11 and 0.30 respectively.

## Discussion

The objectives of this research were to report the magnitude and variation of behavioral changes in calves with ocular injury (corneal scarification), infection and IBK-ACA. The motivation for such information is the need to improve detection of IBK and ocular injury, design studies that assess pain mitigation strategies to reduce animal suffering and decrease production losses associated with disease. In order to do this, it is necessary to first identify behavioral changes associated with ocular pain and sickness behavior, and then design studies with sufficient power to detect meaningful differences in these behaviors. An essential element of sample size calculations is specification of the alternative hypothesis, which describes what is considered a meaningful difference in pain and sickness associated changes in behavior and an estimate of expected variation. This study provides data that can be used for such purposes.

First, our results suggest that a combined index of head-directed behavior has the potential to be used as a measure of ocular pain in calves. This inference comes from the finding that there was a significant time period effect for head-directed behaviors. Specifically, the frequency of head-directed behaviors increased during the scarification time period. If the same data form was used to compare interventions, then these values should be used to determine expected samples sizes for intervention studies. This finding is consistent with those obtained during the mechanical nociception threshold component of this study, as determined by pressure algometry applied to landmarks surrounding the scarified and non-scarified eyes on day -4 and day +1 relative to scarification ( Dewell *et al.*, 2014). Interestingly, mechanical nociception thresholds were affected at all landmarks (surrounding treated and healthy eyes, as well as on the center of the face) suggesting a calf-level change in response, perhaps due to general hyperalgesia associated with the scarification procedure or due to habituation to stressors of handling and restraint. The findings of this behavioral component of the study suggest the former interpretation, since the head-directed behaviors occurred in the home pen when handlers were not present and prior to the post-scarification nociception tests.

Our results are consistent with other studies quantifying behavioral changes associated with head wounds resulting from disbudding and dehorning surgeries. Frequencies of head shaking, head rubbing, and ear flicking by calves increased after hot-iron cautery disbudding surgery relative to behaviors observed after calves received a sham procedure with an unheated dehorner ( Duffield *et al.*, 2010). Behavioral responses were lower in calves that received the nonsteroidal anti-inflammatory drug (NSAID) meloxicam at the time of disbudding relative to calves that received a saline treatment ( Duffield *et al.*, 2010). Similarly, frequencies of head shaking, ear flicking, and head rubbing following cautery disbudding were found to be lower in calves that received ketoprofen versus those that did not receive an NSAID ( Duffield *et al.*, 2010; Faulkner & Weary, 2000). Head rubbing has also been reported when disbudding is performed using caustic paste, and the response was not mitigated by the NSAID flunixin meglumine ( Stillwell *et al.*, 2008). These other studies suggested that the changes in head-directed behavior are not specific to ocular pain. We interpret these changes in behavior as indicators of ocular pain, but it is also possibly due to irritation or itching from the head-restraint or procedure. Calves in our study were examined daily for any physical abnormalities and no lesions associated with trauma from the head-restraint or dermatological conditions of the head were noted. The consistency between behavioral and nociception responses in this study during, before and after scarification, together with the similar consistency in behavioral and nociception responses before and after disbudding which are mitigated when calves are provided with NSAID analgesia ( Duffield *et al.*, 2010), supports the pain interpretation.

We hypothesized that sickness behavior would be expressed by calves that developed IBK lesions, due to cytokines resulting from the inflammatory response. We expected to see decreases in the time spent feeding and standing, together with increases in time spent resting because febrile animals with infections commonly display depression, anorexia, altered grooming patterns and increased time sleeping ( Hart, 1988). These sickness behaviors are presumed to help the body conserve energy and recover from the infection or disease, and are mediated by pro-inflammatory cytokines that act in a paracrine and endocrine manner at the site of inflammation, but also as neurotransmitters that can be produced by glial cells within the central nervous system ( Dantzer & Kelley, 2007). *M. bovis* is a Gram-negative coccobacillus bacterium, and would be expected to produce the classical sickness behavior response ( Postma *et al.*, 2008). There are two primary reasons that could explain why our data did not document an association between sickness behavior and treatment.

First, it is possible the calves were experiencing sickness motivation, but did not demonstrate changes in the variables we measured to indicate sickness behavior. The behaviors selected for our ethogram are consistent with expression of sickness behavior in cattle ( Borderas *et al.*, 2008; Hart, 1987; Proudfoot *et al.*, 2012; Weary *et al.*, 2009). Calves challenged with lipopolysacchride displayed reductions in hay eating, self-grooming and increased duration of lying ( Borderas *et al.*, 2008). However, the small pens in this study limited the behavioral repertoire of the calves, making changes from baseline behavior difficult to observe. Changes from baseline (healthy) behavior to sickness behavior may have been more apparent if calves were housed in larger and more complex pens in which they could interact with more stimuli. For example, play behavior by calves, such as bucking and running, is suppressed after cautery disbudding ( Mintline *et al.*, 2013). Calves were individually housed so they did not compete for access to feed and they were weaned calves so their dam’s influence on behavior was absent. Individual calf variation may potentially explain the lack of increased sickness behavior during scarification time period due to individual variation in regard to temperament, physiology and tolerance. Numerous environmental factors were controlled for in the biosecure research facility, including climactic factors, which may influence the perception of pain, development of IBK and expression of behavior. Further, our study controlled for insect interference because other studies have shown confounding between increase counts of ear flicks following painful procedures when high insect burden is present ( Theurer *et al.*, 2013a). Since calves were housed individually, social facilitation of behavior was less likely, but may have occurred due to auditory and visual contact with neighboring calves. However, since all treatments were housed in a single room and randomly assigned to pens, social facilitation of behavior would not explain differences in behavioral outcomes reported.

Another possible reason why sickness behaviors were not significantly different between time periods was the length of time between onset of inoculation and development of systemic infection. As expected, *M. bovis* calves developed IBK ulcers, but it is possible that the time from scarification to observation was insufficient to detect sickness behavior. Previous experience with this challenge model suggested that *M. bovis* infected animals would develop IBK ( Rosenbusch & Ostle, 1986), and we expected to observe a longer duration of infection (days versus hours) before reaching the IBK ulcer diameter (15mm), which was identified as the humane endpoint for objective of the primary focus of the challenge study ( Gould *et al.*, 2013). In our study some calves developed IBK lesions within four hours of scarification, and others did not develop lesions until 24 hours after scarification. Behavioral changes in calves challenged with *Mannheimia haemolytica,* such as duration of lying, occur from d0 through d+ 8 ( Theurer *et al.*, 2013b), suggesting that a longer interval may have provide more opportunity to observe sickness behavior. However, increases in frequency of occurrence and decreases in duration of lying bouts of cattle infected with the gastrointestinal parasite *Osteragia osteragi* occurred only for animals inoculated at high versus moderate or low doses ( Szyszka & Kyriazakis, 2013). Furthermore, nursery age pigs were found display significant behavioral changes within hours of inoculation with swine influenza virus ( Millman, 2012), suggesting modulation of sickness behavior according to host, agent and environmental factors deserves greater scrutiny.

Continuous video observation was chosen for this study over other options such as scan, time, and focal animal sampling for many reasons. The rationale for this choice, was that continuous video monitoring provided advantages of avoiding the potential for suppression of behavior due to the presence of a human observer in the room, facilitating collection of subtle behavior patterns and rare behaviors that are difficult to capture with instantaneous scan sampling, opportunity for breaks and greater concentration when data are collected over long periods of time, blinding of the observer to the treatments and time period segments to avoid bias, and validation of the ethogram and data collected using inter- and intra-observer reliability tests prior to and during data collection phases. Given that so little information is known about the topic of ocular pain in cattle, the labor and resources were justified. It may be advisable to sample smaller segments of video in subsequent studies to reduce the time-consuming and labor-intensive aspects of continuous scanning. However, we were unable to conduct statistical analyses for some behaviors due to their rarity. Biotelemetry may provide opportunities to collect some behavior automatically, such as with accelerometers attached to a limb to measure bouts of general activity and rest, or more specifically to quantify head only activity if possible affix the device to the calf’s head using a halter or adhesive. If feasible, biotelemetry could reduce the labor associated with behavior data collection substantially.

Although the single observer was trained, misclassification bias is always a possibility in observational studies due to the difficulty in consistently observing the same behavior through multiple videos. If bias did occur due to a single reader, then we hypothesized that the direction of bias was to decrease the frequency of events, i.e. underestimation. That is, while one event was recorded, another may have been missed. This occurrence is an artifact of taking observations off of a quad unit where four unique animals were observed simultaneously, rather than observing one animal from a single camera view. However, we reduced the potential for observation bias by training the observer to a standard kappa compared to a very experienced reader (RP) and to periodically check the intra-reader reliability.

## Conclusion

Our study showed that frequency of head-directed behaviors (head shaking, head rubbing and head scratching) was associated with ocular scarification, and together with changes in nociception thresholds, suggestive of ocular pain. As beef calves are often observed at a distance on pasture and corneas are difficult to evaluate remotely, head rubbing, shaking and scratching, may be early behavioral indicators that producers could use to identify calves to be more closely evaluated for the presence of corneal lesions or other ocular abnormalities. Sickness behaviors were not found to be significantly associated with scarification or IBK ulcers when measured up to 8 h post-scarification. These results describe the magnitude and variability associated with behavioral responses to ocular scarification and IBK ulcers in a challenge model, and can be used for determining sample size calculations for future studies addressing pain mitigation for cattle suffering from ocular injury or disease, such as IBK or for exploring associations between behavior and performance during naturally occurring IBK infections. In conclusion, our research expands the breadth of knowledge for pain and sickness behaviors in cattle, specifically behaviors associated with IBK.

## Data and software availability

Zenodo: Dataset and Code: Pain and sickness behavior associated with corneal lesions in dairy calves. 10.5281/zenodo.18854 ( Woods *et al.*, 2015).
