# Supplementary material for: Pain and sickness behavior associated with corneal lesions in dairy calves
Source: F1000Res. 2015 Aug 11;4:546. [Version 1] doi: 10.12688/f1000research.6649.1 (PMC4758373; doi:10.12688/f1000research.6649.1)
Supplement: Peer review history

# Peer review: Referee response for version 1

**DOI:** 10.5256/f1000research.7143.r12455

**Published:** 2016-02-17

**Authors:** Richard B Evans

**Affiliations:** 1 RCVS Knowledge, London, UK

One reviewer (Dr. Laven) of this article noted an apparent conflict in the statistical reporting. He states,

*There is one statistical misstep, which is repeated on multiple occasions. After demonstrating no effect of treatment or its interaction with time on behaviour, they then compare the baseline and scarification results for each treatment and report that there were treatment effects for the differences. For example, for the combined head behaviours there was no effect of treatment or interaction with treatment and time (p=0.42 and 0.18, respectively); yet it is reported that for control group baseline and scarification were not different (p=0.75) but they were for the two other groups (p=0.02 and 0.04). This is effectively saying there is an interaction between treatment and time when it’s just been shown that there isn’t.  As it’s not really referred to later losing this analysis does not affect the conclusions of the data.*

This is not a misstep. A lack of statistical significance for the interactions means that there wasn’t enough evidence to detect an interaction, not that an interaction doesn’t exist. The interaction test will have lower power than other tests. The authors correctly double checked the group significance cross-sectionally. It’s a good thing they did, because there is a slight interaction effect.

That careful analysis is typical of this paper. The authors methodically verify each step of the study, leaving the reader with little doubt as to the strength of the conclusions. Enough details are provided so that another research team could replicate the study.

I have read this submission. I believe that I have an appropriate level of expertise to confirm that it is of an acceptable scientific standard.

# Peer review: Referee response for version 1

**DOI:** 10.5256/f1000research.7143.r9945

**Published:** 2015-10-13

**Authors:** Richard Laven

**Affiliations:** 1 Institute of Veterinary and Biomedical Sciences, Massey University, Palmerston North, New Zealand

IBK is a common painful condition calves, yet there is limited evidence of its impact on calf behaviour. Previous research by this group has looked at the changes in pain sensitivity associated with ulceration. This paper sets out to look at behavioural changes associated with IBK.

The study is clear and well-planned with three distinct treatment groups (scarification, scarification + apparently non-pathogenic bacteria; scarification + pathogenic bacteria). Unfortunately, as with the previous pain sensitivity study, the impact of the lesions of IBK resulting from infection of a scarified cornea is obscured by the impact of the scarification required as part of the disease-producing model. This limits the value of the study as a study of IBK rather than ocular pain. This is further compounded by the short time interval over which the data were collected.

However, the data collection process is well-planned; in particular, the choice of measures to include in the ethogram is well explained and based on previously published data. The statistics are, for the most part, clearly explained, even though they are complex. One are that was not clear was the choice of model. The paper states “A scaled Pearson’s statistic ( *χ* ^2^ *n/* *df* where *df* is the degrees of freedom) was used to select the preferred modeling approach. We preferred the model with the statistics closer to 1” to which the immediate questions for the non-statistician are how and why?

There is one statistical misstep, which is repeated on multiple occasions. After demonstrating no effect of treatment or its interaction with time on behaviour, they then compare the baseline and scarification results for each treatment and report that there were treatment effects for the differences. For example, for the combined head behaviours there was no effect of treatment or interaction with treatment and time (p=0.42 and 0.18, respectively); yet it is reported that for control group baseline and scarification were not different (p=0.75) but they were for the two other groups (p=0.02 and 0.04). This is effectively saying there is an interaction between treatment and time when it’s just been shown that there isn’t.  As it’s not really referred to later losing this analysis does not affect the conclusions of the data.

The conclusion that head based behaviours could be used to detect early signs of ocular disease is valid, but its utility is reduced by the problems with the model of producing the disease as all of the changes found in this study are really a response to the scarification required to produce corneal damage that can then become infected by *M. bovis*.  A better model with a less painful initial stage would be useful – perhaps introducing naïve calves to a group of previously infected cattle (either naturally or artificially induced); this should be discussed in the paper.

I have read this submission. I believe that I have an appropriate level of expertise to confirm that it is of an acceptable scientific standard.
